# Supplementary material for: Identification of Freezing-Responsive microRNAs and Their Targets in Chinese Jujube by Small RNA and Degradome Sequencing
Source: Int J Mol Sci. 2026 Sep 4;27(17):7912. doi: 10.3390/ijms27177912 (PMC13566890; doi:10.3390/ijms27177912)
Supplement: Supplementary file 1 [file ijms-27-07912-s001.zip › Supplementary Material S2/novel_miR_95.pdf]

Provisional ID	:	novel_miR_95
Score total	:	0.3
Score for star read(s)	:	-1.3
Score for read counts	:	0
Score for mfe	:	0
Score for randfold	:	1.6
Score for cons. seed	:	
Total read count	:	93
Mature read count	:	93
Loop read count	:	0
Star read count	:	0

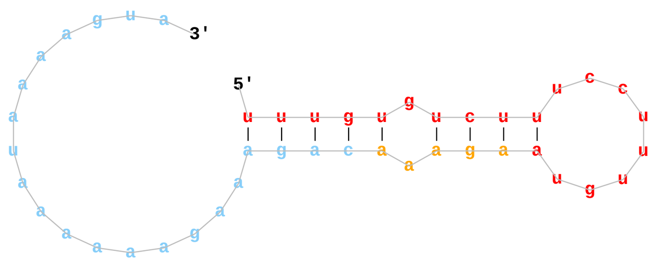

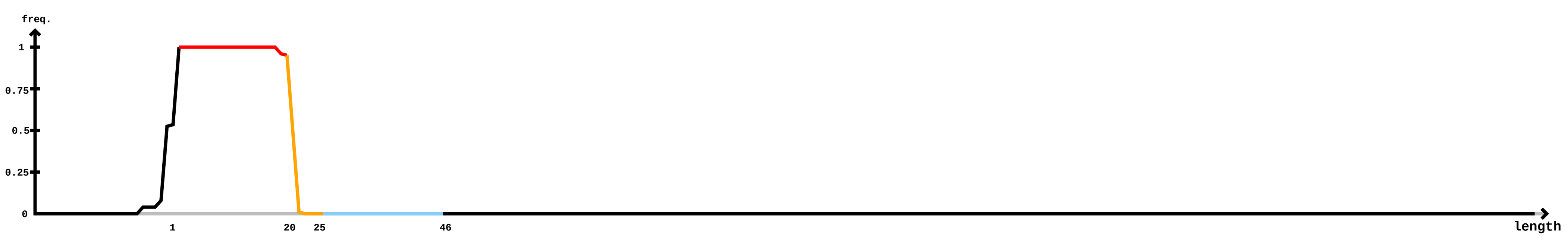

Mature Star

```
5'- caaaaaaaaauuucuccuaaauauuugugucuuuccuuuguaagaaacagaaagaaaaaauaaaaguauuagagagagacaaagaaaauaaagaaauagaaaaugaaaguauuaaugggaaaaaaaaaauauuuucuaaauacuuguguauuucuuuuguuaaaaagaguguuuuucuccuauuauaaaagaaaugauauugucaacuaugcucuauuuauuuuaaagagaaaucauuauauucuguuacu -3'   exp
    .........(((((((((.(((((.(((.((((.(((((.....)))))..))))..))).))))))))).))))))...........((.(((((((.(((((((((((..(((((((........))))))))))))))).((.(((((((((..((((.(((((((.......((((((......)))))).........))))))).)))).....))))))))))))))).))))))).))   reads   mm   sample
    ................uaaauaCuugugucuuuccuuugua..............   4    1   LCK-3
    ...................auauuugugucuuuccuuuA................   3    1   LT-3
    ...................auauuugugucuuuccuuug................   1    0   LT-3
    ....................uauuugugucuuuccuuugua..............   21   0   LCK-1
    ....................uauuugugucuuuccuuugua..............   23   0   LCK-2
    ....................uauuCgugucuuuccuuuguaag............   1    1   LT-2
    .....................auuugugucuuucUuuugu...............   1    1   LCK-3
    ......................uuugugucuuuccuuugua..............   22   0   LCK-2
    ......................uuugugucuuuccuuugua..............   25   0   LCK-1
```
